# TAS-102, the first “cardio-gentle” fluoropyrimidine in the colorectal cancer landscape?

**DOI:** 10.1186/s12885-016-2409-8

**Published:** 2016-07-04

**Authors:** Fausto Petrelli, Sandro Barni, Paola Bertocchi, Alberto Zaniboni

**Affiliations:** Department of Oncology, Division of Medical Oncology, Azienda Ospedaliera Treviglio, Piazzale Ospedale 1, 24047 Treviglio, BG Italy; Oncology Department, Fondazione Poliambulanza, Via Bissolati 57, 25100 Brescia, Italy

**Keywords:** TAS-102, Cardiotoxicity, Fluoropyrimidines

## Abstract

**Background:**

Cardiotoxicity in the form of cardiac arrhythmia, myocardial infarction, and angina-like symptoms are not rare complications of fluoropyrimidines as 5-Fluorouracil (5FU) and capecitabine.

**Discussion:**

Tas-102, a novel oral fluoropyrimidine, was recently approved by FDA for the treatment of advanced and refractory colorectal cancer. Its unique mechanism of action doesn’t seem linked with cardiotoxicity in clinical trials reported so far.

**Summary:**

TAS 102 may represent one of the drugs of choice for patients with advanced colorectal cancer with cardiac disease. This intriguing and clinically relevant issue is briefly examined.

## Background

Cardiotoxicity in the form of cardiac arrhythmia, myocardial infarction, and angina-like symptoms are not rare complications of fluoropyrimidines as 5-Fluorouracil (5FU) and capecitabine (Table 1). The hallmark of cardiotoxicity is the occurrence of coronary vasospasm leading to the electrocardiographic and clinical manifestation of myocardial ischemia. Meyer et al. [1], in a prospective series of more than 400 patients treated with 5FU continuous infusion (ci), found an incidence of 3 % (9 cases out of all observed patients) during the first course of chemotherapy, and this was one of the first association of type of 5FU administration and risk of cardiotoxicity, with preexisting cardiac disease and concomitant cardiovascular drugs as main risk factors in these 9 patients. From that publication, other series found evidence of cardiac events occurrence during the administration of 5FU ci (or capecitabine) for gastrointestinal malignancies. In particular, myocardial angina or cardiac infarction are the most observed clinical manifestation of 5FU-related cardio-toxicity, and other antimetabolites (e.g., raltitrexed) or bolus 5FU administration could mitigate the risk of these worrisome events [2, 3]. The exact pathophysiology of these events is not well known, but a cardiac vasospasm seems the main events associated with cardio- toxicity. It has been postulated a role of the 5FU metabolites: in particular 5FU is catabolized to alpha-fluoro-beta-alanine (FBAL) and subsequently to fluoroacetate, the latter suspect in particular as a cardiotoxic substance [4]. Recently, Italian authors analyzed human cardiomyocytes and endothelial cells to evaluate the effect of 5FU. They observed autophagic features with reactive oxygen species (ROS) elevation as an endothelial response and induction of a senescent phenotype on both cell types treated with 5FU [5]. By these data and owing to the relative misunderstanding of exact mechanisms involved in the cardio-toxicity of 5FU, there is a rationale for using different inhibitors of thymidylate synthase (TS), commonly prescribed in clinical practice, in particular, raltitrexed. Animal studies of myocardium damage after 5FU administration showed that multifocal hemorrhages, myofiber necrosis, inflammatory reactions including perivascular involvement, pericarditis, valvulitis and vascular changes, were observed. In particular, a single high intravenous dose resulted in hemorrhagic infarction of the ventricle walls, proximal spasms of the coronary arteries and lethal outcome for animals within few hours. In contrast, repeated lower doses resulted in left ventricular hypertrophy, concentric fibrous thickening of the intima of coronary vessels and foci of necrotic myocardial cells [6]. Whether the differences in histopathological effects were due to different doses, is not understood but could be the basis for an oral (chronic) low dose of antimetabolites as TAS 102 and S-1.

**Table 1:** Cardiotoxicity of capecitabine, 5-Fluorouracil, TAS 102 and S1

	Very common	Common	Uncommon	Rare
Capecitabine			Unstable angina pectoris, angina pectoris, myocardial ischemia, atrial fibrillation, cardiac arrhythmia, tachycardia, Palpitation	Ventricular fibrillation, QT prolongation, torsade de pointes, bradycardia, vasospasm
5-fluorouracil		Chest pain	Myocardial infarction, myocardial ischemia, cardiac arrhythmia, myocarditis, heart failure, dilated cardiomyopathy	Cardiac arrest, sudden cardiac death
TAS-102				Myocardial infarction, myocardial ischemia, chest pain, bradycardia, tachycardia
S1			Cardiac failure, acute myocardial infarction, pericardial effusion, atrial fibrillation, angina pectoris, cardiac fibrillation, tachycardia, palpitations	

## Main text

**Fig. 1:**
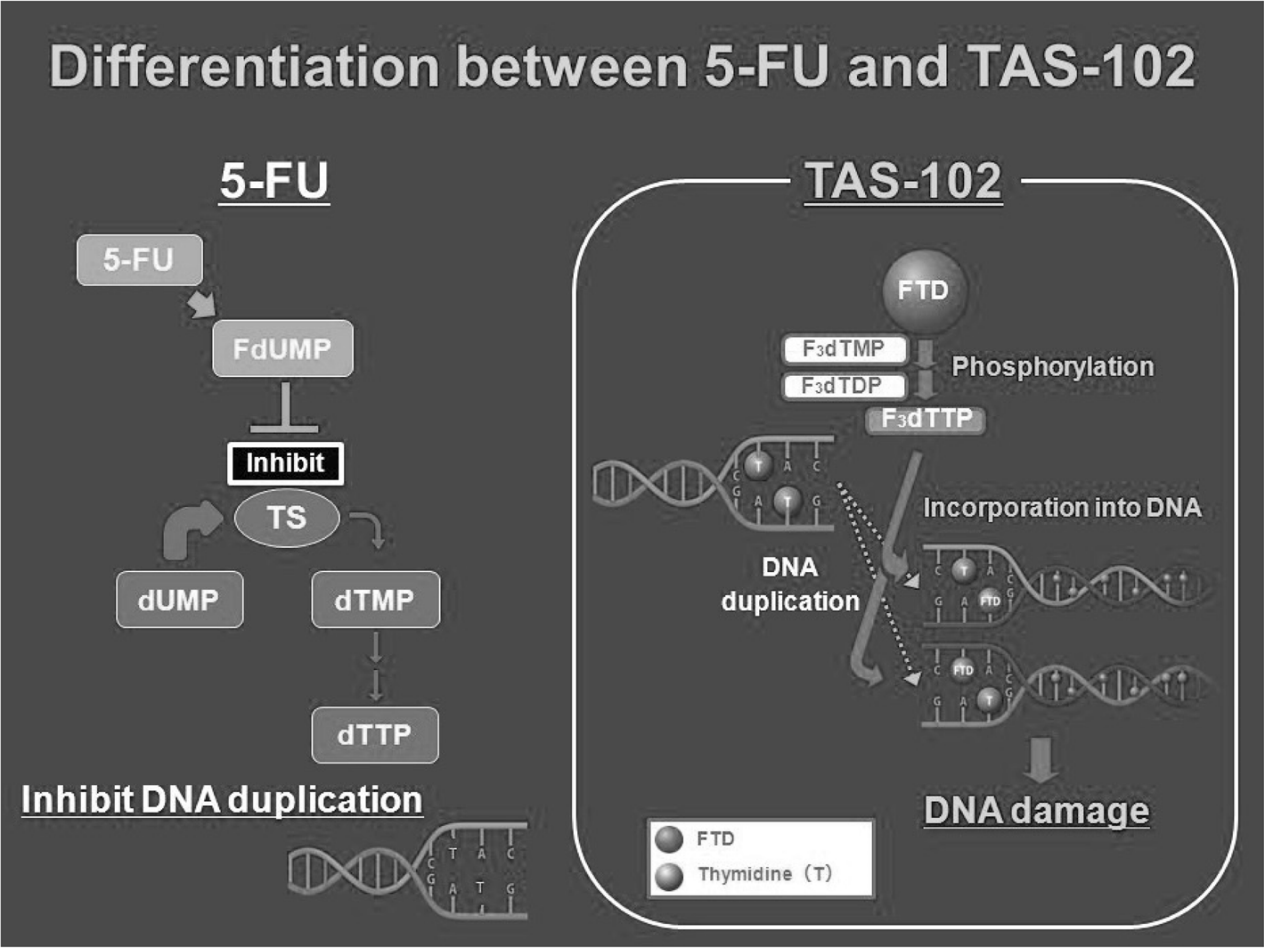
TAS 102 mechanism of action compared to 5-FU

TAS-102 is a new, recently labeled, oral fluoropyrimidine used for chemorefractory colorectal cancer. It increased overall survival by 2.4 months in patients with metastatic colorectal cancer, pretreated with all active drugs and biologics [7]. It has been developed in Asiatic countries, and it has recently been approved in this setting by FDA. TAS-102 consists of tri-fluorothymidine (TFT) and an inhibitor of thymidine phosphorylase (TPI). TFT, an analog of thymidine, exhibits two mechanisms of anti-tumour actions: inhibition of TS, similarly to the mechanism of action of 5FU, and the creation of single-strand DNA breaks by incorporating the triphosphate form of TFT into DNA (Fig. 1). The concomitant association of TFT and TFT and TPI (5-chloro-6- (2-iminopyrrolidin-1-yl) methyl-2, 4 (1H 3H) -pyrimidinedione hydrochloride) showed an increase in the plasma concentrations and anti-tumour activity of TFT for inhibition of TFT degeneration [8]. TAS-102 is an oral anti-cancer drug consisting of TFT and TPI combined at a molar ratio of 1: 0.5. The labeled schedule of TAS 102 is 35 mg/m^2^ given twice a day in a 28-day cycle orally (2-week cycle of 5 days of treatment followed by a 2-day rest period, and then a 14-day rest period).

We searched for phase I, phase II and randomized phase III trials to evaluate if cardiovascular events were reported and if any, which type of toxicity was observed [7, 9–13]. Only studies reporting the current, labeled scheduled of TAS 102 were considered. Among 4 phase I studies, no cardiotoxic events were reported, including 3 single agent TAS 102 trials and a phase I study of a combination of TAS 102 with a fixed dose of irinotecan (121 patients overall). In the phase II trials comparing TAS 102 (at the labeled dose) with placebo, 112 patients were randomized but no cardiotoxic events were reported yet. Finally in the pivotal phase III study by Mayer et al. *n* = 3 and *n* = 2 cardiac events (cardiac ischemia) were recorded in the experimental and control arms respectively, with no rate of cardiac arrhythmia observed. The rate of cardiotoxicity in this trial is 0.5 % that represents from 2 to 40-fold numerically less frequent of that reported with 5FU in literature. A potential limitation of our observation is that the patients who had cardiotoxicity during previous treatments might have been excluded from TAS102 trials, which enrolled patients heavely pretreated with many previous lines of therapies. Nevertheless, we think that the different mechanism of action of TAS 102 compared with others fluoropyrimydines might still explain the lack of cardiotoxicity reported so far with this novel drug.

Different putative mechanisms of cardiotoxic-free events in patients treated with TAS 102 could be postulated:

1. A different autophagic induction of TAS 102 compared to 5FU. In Focaccetti et al. paper cited above, autophagic features at the ultrastructural and molecular levels, in particular in 5-FU exposed cardiomyocytes and endothelial cells can be observed. Conversely, TFT was more potent than 5FU, because it induces higher levels of cell death and does not elicit an autophagic survival response in the cancer cell lines [14].
2. A different oncological target (DNA damage and synthesis) for TAS 102 itself compared to 5FU or capecitabine: TF-TMP (tri-fluorothymidine monophosphate) and TF-TTP (tri-fluorothymidine triphosphate) vs FdUMP (fluorodeoxyuridine monophosphate) and FdUTP (fluorodeoxyuridine triphosphate).
3. The incorporation of FTD into DNA in tumor tissues is significantly higher than its incorporation into DNA in normal tissues, thus sparing cardiovascular tissues [15].
4. TAS 102 is not catabolized by dihydropyridine dehydrogenase (DPD), that is predominantly expressed in the liver and inactivates more than 80 % of administered 5FU, with consequently reduced the formation of FBAL [8]. This is somewhat similar to that happen with S-1 that is a combination of tegafur, gimeracil, and OXO. Because of the significant DPD inhibition by gimeracil, FBAL and other cardiotoxic 5FU catabolites (F-citrate) levels are significantly lower than after capecitabine or I.V. 5-FU administration, and less cardiotoxicity can be expected [16]. F-citrate inhibits the Krebs cycle by inhibition of aconitase, limiting the cellular production of adenosine triphosphate (ATP). Fluoroacetate is known to be highly cardiotoxic and neurotoxic [17].

## Conclusions

We can reasonably affirm that the new oral agent TAS 102, represent one of the true drugs of choice for patients with advanced colorectal cancer with known (pre-existing) cardiac disease or for those which manifest cardiac complications during 5FU or capecitabine treatment. It has several potential advantages over raltitrexed (oral route and less renal toxicity) and S-1 that is not still labeled for colorectal cancer in western countries. Another possible benefit of TAS 102 is the possibility of administering the drug to those subjects with known DPD deficiency, so reducing the expected risk of severe gastrointestinal toxicity. Combination studies with other cytotoxics are in fiery, including biological agents. Hematological toxicities represent the limiting event, but with very low rate of febrile neutropenia. In the meanwhile TAS 102 appear as one the most cardio-gentle nucleoside analog available in clinical practice and may represent the agent of choice when 5FU is indicated but relevant comorbidities increase the cardiovascular risk.

## Abbreviation

5FU, 5-Fluorouracil; ATP, adenosine triphosphate; ci, continuous infusion; DPD, dihydropyridine dehydrogenase; FBAL, alpha-fluoro-beta-alanine; FdUMP, fluorodeoxyuridine monophosphate; FdUTP, fluorodeoxyuridine triphosphate; ROS, reactive oxygen species; TFT, tri-fluorothymidine; TF-TMP, tri-fluorothymidine monophosphate; TF-TTP, tri-fluorothymidine triphosphate; TPI, thymidine phosphorylase; TS, thymidylate synthase
